# Evaluating the Aquatic Environment as a Reservoir for *Salmonella*: A Comparative Analysis with Clinical Strains

**DOI:** 10.3390/microorganisms13092072

**Published:** 2025-09-05

**Authors:** Si Hyun Kim, Gyung-Hye Sung, Eun Hee Park, Suk Nam Hwang, Eun-Young Kim, Eunkyoung You, Ja Young Lee, Gyu Ri Kim, Joseph Jeong, Sunjoo Kim, Jeong Hwan Shin

**Affiliations:** 1 Department of Biomedical Laboratory Science, Inje University, Gimhae 50834, Republic of Korea; 2 Busan Institute of Health and Environment, Busan 46616, Republic of Korea; 3 Ulsan Health and Environment Research Institute, Ulsan 44642, Republic of Korea; 4 Department of Laboratory Medicine, Inje University College of Medicine, Busan 47392, Republic of Korea; 5 Paik Institute for Clinical Research, Inje University, Busan 44392, Republic of Korea; 6 Department of Biomedical Laboratory Science, Kyungwoon University, Gumi 39160, Republic of Korea; 7 Department of Laboratory Medicine, University of Ulsan College of Medicine, Ulsan University Hospital, Ulsan 44033, Republic of Korea; 8 Department of Laboratory Medicine, Gyeongsang National University College of Medicine, Jinju 52727, Republic of Korea

**Keywords:** *Salmonella*, stream, aquatic environment, PFGE, WGS

## Abstract

Aquatic environments are potential reservoirs for the persistence and spread of pathogenic bacteria. This study investigated the prevalence of *Salmonella* spp. in stream environments and their relationship with clinical isolates in Republic of Korea. A total of 4582 water samples were collected from 94 streams. We identified these isolates using MALDI–TOF MS and the Kauffmann–White scheme. Polymerase chain reaction and sequencing were performed to identify the resistance genes. Whole genome sequencing analysis and pulsed-field gel electrophoresis (PFGE) were performed to investigate genetic relatedness. In total, 110 *Salmonella* isolates showing 23 serotypes were isolated from the streams. *S.* Typhimurium (20.9%) was the most common, followed by *S.* Livingstone (17.3%), *S.* Infantis (10.9%), *S.* Othmarschen (6.4%), *S.* I. 4,[5],12:i:- (5.5%), and *S.* Thompson (5.5%). PFGE patterns of eight serotypes were identical or closely related to the stream and clinical strains. The sequence types of *S.* Mbandaka and *S.* Livingstone isolates from streams were identical to those of the clinical specimens as ST413 and ST543, respectively. *Salmonella* strains are highly prevalent in streams and are closely related to the isolates obtained from patients. Therefore, the continuous monitoring of stream environments is required to control the spread of *Salmonella*.

## 1. Introduction

*Salmonella* is a motile, rod-shaped, Gram-negative bacterium that is classified into two species: *S. enterica* and *S. bongori.* Of these, most clinical isolates are *S. enterica* strains, which is divided into six subspecies based on its biochemical characteristics. *Salmonella* strains are composed of more than 2500 serotypes, which are determined by the somatic O and flagellar H antigens [1,2]. It is important to distinguish serotypes because different serotypes exhibit distinct host specificities, virulence traits, and antimicrobial resistance patterns. *Salmonella* is one of the most important pathogens that cause foodborne infections with several symptoms, such as diarrhea, fever, and vomiting. It is well known that *Salmonella* serovar Typhi (*S.* Typhi) and *S.* Paratyphi cause typhoid and paratyphoid fever, respectively, leading to high morbidity and mortality worldwide. In addition, an increase in non-typhoidal *Salmonella* (NTS), such as *S.* Enteritidis, *S.* Typhimurium, and *S.* I 4,[5]12:i:-, is a major public health problem [1,2]. The United States Centers for Disease Control and Prevention (US CDC) estimates that NTS causes approximately 150 million illnesses and 60,000 deaths globally each year [3]. *Salmonella* infections, especially by NTS, are also occurring steadily in Republic of Korea [1]. In addition, the treatment of *Salmonella* infections has become complicated due to the emergence of antimicrobial resistance strains. The global burden of *Salmonella* infections highlights the need for control strategies and continuous surveillance.

Transmission of *Salmonella* to humans occurs mainly through animal products contaminated with *Salmonella*, including meat and eggs. The emergence of foodborne infection with *Salmonella* is a major concern worldwide [4]. Recently, there has been an increase in foodborne disease outbreaks caused by non-traditional sources, such as fresh fruits and vegetables [5,6], with surface water being an important source of raw food contamination. Water is a potentially persistent environmental reservoir for *Salmonella* [7,8]. In the One Health approach, aquatic environments around urban areas are closely related to humans, animals, and other environments, because they are easily contaminated by livestock, human waste, and/or hospital wastewater [9]. Few reports have investigated the role of linking the environment to clinical isolates, although the aquatic environment is increasingly recognized as a reservoir for *Salmonella,* causing human infection. This study aimed to investigate the prevalence of *Salmonella* in stream environments, and its association with various clinical strains.

## 2. Materials and Methods

### 2.1. Collection and Identification of Strains

We collected 4582 water samples from 218 sites in 94 streams in two metropolitan cities (Busan and Ulsan) and one province (Gyeongsangnam-do) in Republic of Korea between July 2017 and August 2019, as previously described [10]. To ensure consistency, sampling was performed equally across three different institutions, following standardized protocols. A fixed number of stream water samples were collected monthly from various sites in each region to maintain a uniform sampling range.

To isolate microorganisms from the water samples, we used the filter paper method with sterile 0.45 μm membrane filters (Merck Millipore, Billerica, MA, USA). After passing water samples through membrane filters, the surface of the filtered membrane was scratched using a sterile cotton swab and cultured on a CHROMagar^TM^ Salmonella Plus plate (CHROMagar, Paris, France) at 35 °C for 48 h. Each *Salmonella*-suspicious colony grown on the medium was sub-cultured on a blood agar plate at 35 °C for 16 h. The colonies were identified using VITEK MS (BioMerieux, Marcy I’Etoile, France). All strains identified as *Salmonella* using the VITEK MS system were serotyped using the White–Kauffmann Scheme. Slide and tube agglutination tests were conducted using antiserum (Becton Biosciences, Franklin Lakes, NJ, USA) for somatic and flagellar antigens, respectively.

All clinical strains of *Salmonella* collected from three university hospitals in Busan, Ulsan, and Gyeongsangnam-do, Republic of Korea, during the same period were included in this study. We determined the serotypes of clinical *Salmonella* strains and compared them with those from stream environments.

### 2.2. Antimicrobial Susceptibility

Antimicrobial susceptibility tests were performed using Sensititre EUVSEC susceptibility MIC plates (TREK Diagnostic Systems/Thermo Fisher Scientific, Cleveland, OH, USA). Ampicillin, chloramphenicol, trimethoprim-sulfamethoxazole (SXT), cefotaxime, ciprofloxacin, azithromycin, tetracycline, and imipenem were used as the antimicrobial agents. The results were interpreted by the guidelines of the Clinical and Laboratory Standard Institute (CLSI M100 34th ed [11]). *Escherichia coli* ATCC 25922 was used as the quality control strain.

### 2.3. Determination of Antimicrobial Resistance Genes

Extended-spectrum beta-lactamase (ESBL) genotyping was performed when *Salmonella* showed resistant or intermediate results for third generation cephalosporin. All isolates were studied by PCR and sequencing of the *bla*_CTX-M_ gene using primer sets, as previously described [12]. We also investigated the presence of mutations in the quinolone resistance-determining region (QRDR) in ciprofloxacin-intermediate and resistant *Salmonella* strains by amplifying and sequencing *gyrA*, *gyrB*, *parC*, and *parE* genes [13,14].

### 2.4. Analysis of PFGE and WGS

We performed pulsed-field gel electrophoresis (PFGE) by selecting strains from serotypes detected simultaneously in stream and clinical samples. We used a standardized PluseNet Salmonella protocol for PFGE and BioNumerics software v.4.00 (Applied Maths, Sint-Martens-Latem, Belgium). The restriction enzyme was Xba I (Roche, 40 U/ul. Basel, Switzerland), and the molecular weight marker was *Salmonella* serovar Braenderup ATCC BAA-664. We considered a strain to be closely related when the similarity of the PFGE dendrogram was ≥90%. We compared the similarity of the strains from the stream with those from clinical samples.

Whole genome de novo sequencing (WGS) was performed at Macrogen (Seoul, Republic of Korea) using the Illumina platform HiSeq4000 system (Illumina, San Diego, CA, USA) with the Nextera Mate Pair Sample Prep Kit (Illumina, USA) and TruSeq DNA PCR-Free kit (Illumina, USA). Sequencing reads were assembled de novo using SPAdes (v3.12), followed by error correction with Illumina short-read data using Pilon (v1.23). Long reads were scaffolded using SOAPdenovo 2 and gaps were filled using GapCloser v1.12. The genome sequences were annotated using Prokka (v1.12). The genome sequences of 9 *Salmonella* spp. isolates generated in this study have been deposited in the NCBI GenBank database (https://www.ncbi.nlm.nih.gov/genbank/ accessed on 18 August 2025). All sequence data generated in this study have been available under BioProject accession number PRJNA1307790. Individual isolate information is available under BioSample accession numbers SAMN50675129–SAMN50675137. The corresponding whole-genome shotgun assemblies have been assigned under GenBank accession numbers JBQQUO000000000–JBQQUW000000000.

The data analysis of whole-genome sequencing (WGS) was performed at the Center for Genomic Epidemiology (CGE) (https://www.genomicepidemiology.org/services/) (accessed on 6 June 2025). This analysis was performed to confirm the genetic relatedness of the two serotypes, which showed 100% similarity between the stream and clinical strains using PFGE. Multilocus sequence typing (MLST) and cgMLST analyses were conducted based on the genomic sequences by analyzing seven housekeeping gene loci: *aroC*, *dnaN*, *hemD*, *hisD*, *purE*, *sucA*, and *thrA*. Multi-Locus Sequence Typing (MLST) and Core Genome Multi-Locus Sequence Typing (cgMLST) were determined by using WGS with MLST-2.0 and cgMLSTFinder-1.2 provided by CGE. Digital DNA:DNA hybridization (dDDH) was analyzed using Type (Strain) Genome Server (TYGS, https://tygs.dsmz.de/).

## 3. Results

### 3.1. Characteristics of Salmonella Serotypes in Streams

A total of 110 *Salmonella* strains (2.4%) were isolated from 4582 water samples. During the study period, 34, 27, and 49 strains were isolated in 2017, 2018, and 2019, respectively. The 110 isolates were divided into 23 serotypes. The most common serotype was *S.* Typhimurium (N = 23, 20.9%), followed by *S.* Livingstone (N = 19, 17.3%), *S.* Infantis (N = 12, 10.9%), *S.* Worthington (N = 8, 7.3%), *S.* Othmarschen (N = 7, 6.4%), *S.* I 4,[5]12:i:- (N = 6, 5.5%), *S.* Thompson (N = 6, 5.5%), and others (N = 29, 26.4%). Seven common serotypes accounted for 73.6% of isolates (Table 1).

In total, 152 clinical strains were isolated from three university hospitals during the same period. *S.* I 4,[5]12:i:- (N = 21, 13.8%) was the most common, followed by *S.* Bareilly (N = 19, 12.5%), *S.* Enteritidis (N = 18, 11.8%), *S.* Thompson (N = 13, 8.6%), *S.* Typhimurium (N = 11, 7.2%), *S.* Agona (N = 11, 7.2%), *S.* Typhi (N = 9, 5.9%), and *S.* Infantis (N = 8, 5.3%); these serotypes accounted for 72.4% of the isolates.

**Figure 1:**
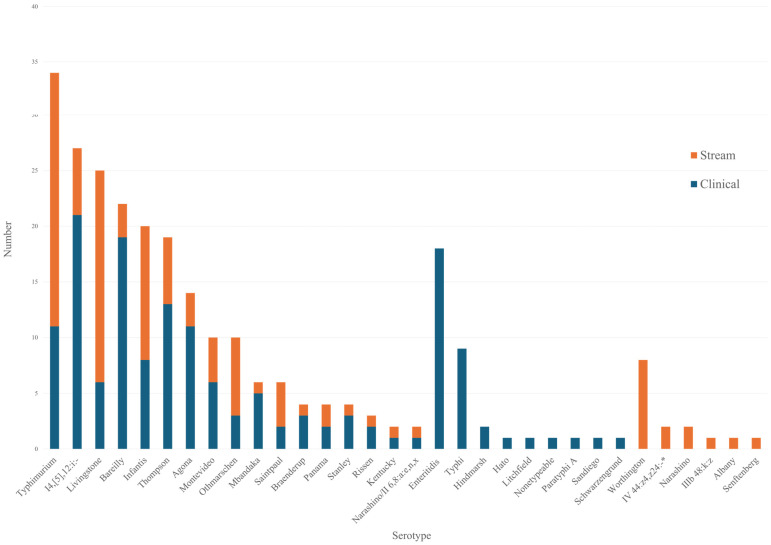
Serotype distribution between stream and clinical isolates. * IV 44:z4,z24:/Christiansborg/IIIa 44:z4,z24:-.

Among the 23 serotypes isolated from the stream, 17 were also detected in clinical samples (Figure 1). The highly prevalent serotypes of *Salmonella* from streams were also frequent in clinical samples, including *S.* Typhimurium, *S.* Livingstone, and *S.* Infantis. *S.* Enteritidis and *S.* Typhi were not detected in the streams, although they are common clinical strains. *S.* Worthington was isolated with the fourth highest frequency in stream environments, whereas it was not detected in clinical samples. *S.* Narashino, *S.* IV 44:z4,z24:-/Christiansborg/IIIa 44:z4,z24:-, S. IIIb 48:k:z, *S.* Albany, and *S.* Senftenberg were also isolated only from stream samples.

### 3.2. Antimicrobial Resistance and Molecular Characterization of Resistance Genes

The antimicrobial resistance of *Salmonella* strains isolated from the streams is presented in Table 2. The rates of resistance to ampicillin, chloramphenicol, and SXT were 27.3%, 19.1%, and 20.9%, respectively. All *Salmonella* strains were susceptible to azithromycin and imipenem. Almost all *Salmonella* strains were susceptible to cefotaxime, except for one (*S.* Albany). No CTX-M-type ESBL genes were detected.

The resistance rates of clinical isolates to ampicillin, chloramphenicol, and tetracycline were 19.3%, 10%, and 17.7%, respectively. All *Salmonella* strains were susceptible to azithromycin and imipenem. The clinical isolates showed a slightly higher resistance rate to cefotaxime at 2.4% than stream isolates; however, the stream isolates exhibited a higher resistance rate to SXT than clinical isolates.

Although none of the strains were resistant to ciprofloxacin, the intermediate resistance rate was as high as 26.4% (N = 29). In clinical strains, the ciprofloxacin resistance rate was 2.4% and the intermediate resistance rate was 20.5%. Of the 29 strains from the stream, 10 harbored mutations in *gyrA* and *parC* (Table 3). In *gyrA*, D87N was the most frequent QRDR mutation, and all strains were *S.* I 4,[5]12:i:- (N = 6). Four strains showed a mutation pattern of T57S in parC, and two of these four strains carried a *gyrA* mutation (D87G or S83F). None harbored mutations in *gyrB* and *parE*.

### 3.3. Epidemiologic Association of Salmonella from Stream and Clinical Samples

**Figure 2:**
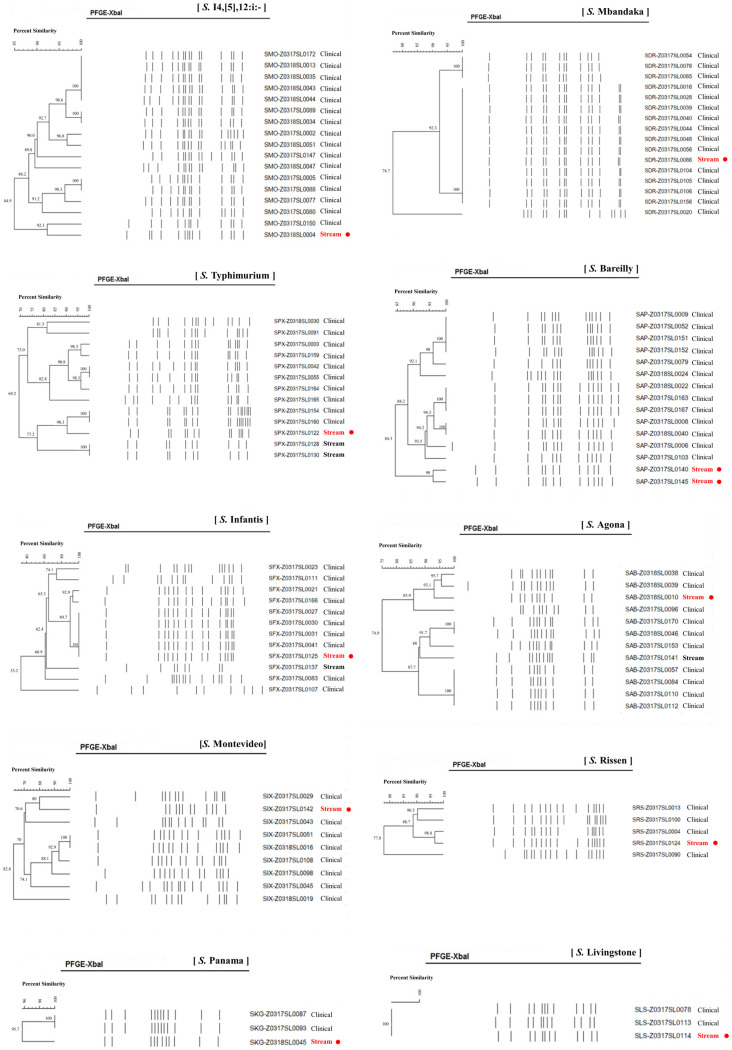
PFGE patterns of *S.* I 4,[5],12:i:-, *S.* Mbandaka, *S.* Typhimurium, *S.* Bareilly, *S.* Infantis, *S.* Agona, *S.* Montevideo, *S.* Rissen, *S.* Panama, and *S.* Livingstone. Red points indicate Salmonella strains from stream are closely related with those from clinical specimen. Black bolds indicate Salmonella strains from stream are not related with those from clinical specimen.

We performed PFGE on 10 serotypes of 103 *Salmonella* strains, consisting of 23 stream and 80 clinical samples: *S.* I 4,[5],12:i:-, *S.* Typhimurium, *S.* Mbandaka, *S.* Livingstone, *S.* Bareilly, *S.* Montevideo, *S.* Agona, *S.* Infantis, *S.* Rissen, and *S.* Panama (Figure 2). Serotypes *S.* Mbandaka, *S.* Infantis, and *S.* Livingstone showed 100% PFGE homology between stream and clinical strains. Similarly, *S.* I 4,[5],12:i:-, *S.* Typhimurium, *S.* Agona, *S.* Rissen, and *S.* Panama were closely related, with 92.3%, 90.3%, 93.1%, 96.8%, and 95.7% similarities between the stream and clinical strains, respectively. We found a correlation between the stream and clinical strains in the *S.* Bareilly and *S.* Montevideo of 84.3% and 80.0%, respectively.

WGS was performed for two serotypes of *S.* Mbandaka (stream = 1, clinical = 5) and *S.* Livingstone (stream = 1, clinical = 2), showing 100% similarity between stream and clinical strains by PFGE (Table 4). *S.* Infantis was excluded because the regions separating the streams and hospitals were different. We found >4,600,000 bp of genomic sequences, 7–13 different contigs, and 4300 genetic sequences encoding proteins. All *S.* Mbandaka strains showed 100% homology to each other in the dDDH analysis, except for one (99.8%). In terms of G + C content, all serotype *S.* Mbandaka strains were 100% consistent, excluding one strain that showed a difference of 0.16% from the other strains. For the three strains identified as *S.* Livingstone, the dDDH values showed 100% homology.

The MLST type of all six *S*. Mbandaka strains, including the stream and clinical strains, was ST413. The cgMLST type confirmed that all strains matched ST110743, except for one clinical strain (ST123743). For *S.* Livingstone, the MSLT and cgMLST types of isolates from stream were the same, identified as ST543 and ST73367, respectively, and identical to clinical isolates.

## 4. Discussion

The transmission of *Salmonella* to humans is associated with various environmental systems, including animals, food, and water [15]. The aquatic environment, which is a vital resource for human and animal survival, is an important route of contamination [8]. Water sources contaminated by foodborne pathogens lead to intestinal infection through fresh produce, such as fruits and vegetables. Several studies have reported the presence of *Salmonella* in diverse aquatic environments. Baudart et al. isolated >40 *Salmonella* serotypes from various natural aquatic systems in France [16]. Similarly, Callahan et al. [8] detected *S. enterica* in river waters of the Maryland Eastern Shore, USA, and Jimenez et al. [17] observed a high persistence of *Salmonella* within rivers of the Culiacan Valley, Mexico. The 2005 multistate outbreaks of *Salmonella* suggest the potential for persistent contamination of tomato fields, with the same *S.* Newport stain found in pond water used for irrigation [18]. These findings highlight the role of the aquatic environment in pathogen transmission and the importance of identifying these relationships to effectively control disease outbreaks.

This study reveals the presence of diverse *Salmonella* strains in Republic of Korean streams. Notably, the 110 isolates comprised 23 distinct serotypes, including *S.* Typhimurium, *S.* Livingstone, *S.* Infantis, *S.* Worthington, *S.* Othmarschen, *S.* I. 4,[5]12:i:-, and *S.* Thompson. However, there are some differences in the dominant serotypes among several countries. For instance, in Delaware, the United States, *S.* Newport, *S.* Typhimurium, *S.* 4,[5],12: i:-, *S.* Bareilly, *S.* Enteritidis, and *S.* Given were the predominant and seasonally persistent serotypes between 2015 and 2016 [8]. A study in France reported that *S.* Typhimurium was dominant in all river sites, whereas *S.* Newport was mostly isolated from wastewater samples [16]. In addition, a five year study (2011–2016) on the Central California coast showed the dominance of *S.* I 6,8:d:-, *S.* Give, *S.* Muenchen, *S.* Typhimurium, *S.* Oranienburg, and *S.* Montevideo [19]. Although there is regional variation, we confirmed that *S.* Typhimurium is the most frequently isolated serotype in water samples. *S.* Typhimurium and *S.* Enteritidis have been the most prevalent serotypes in Korea over the past 10 years. *S.* Typhimurium is the third most common serotype of human salmonellosis in the USA [20]. Interestingly, 17 of the 23 serotypes detected in the streams in this study were also detected in human specimens from Korea [1]. Furthermore, PFGE results showed high similarity (>90%) for the eight serotypes between the stream and clinical strains. These findings suggest that *Salmonella* infection in humans may be closely related to the presence of *Salmonella* in streams.

On the other hand, some differences were also observed in the serotype distribution between stream and clinical strains. Serotypes of Worthington, IV 44:z4,z24:-/Christiansborg/IIIa 44:z4,z24:-, Narashino, IIIb 48:k:z, Albany, and Senftenberg were only isolated from stream samples. However, these serotypes, except for *S.* Worthington and *S.* IV 44:z4,z24:-/Christiansborg/IIIa 44:z4,z24:-, were also detected in clinical samples, as confirmed by our previous nationwide surveillance study [1]. *S.* Worthington was very common in the stream. There are several reports of outbreaks in neonates, and it causes sepsis and meningitis [21]. Although it is hardly seen in Republic of Korea from clinical isolates, it is important to monitor continuously because of its potential pathogenicity. While serotypes of Enteritidis, Typhi, Paratyphi A, Hindmarsh, Hato, Litchfield, Sandiego, and Schwarzengrund were only isolated from clinical samples, *S.* Enteritidis and *S.* Typhi were isolated at high frequency in humans. However, these were not found in stream samples. Unlike our findings, *S.* Enteritidis was recovered from the river in France and the United States, and this serotype is the most common serotype in these two countries in clinical samples [8,16]. We could not confirm why these serotypes were not seen in the stream in our study. The factors contributing to *Salmonella* contamination in the stream environment may be diverse, and it can originate from human wastewater, livestock effluent, and other environmental inputs. In addition, human infections are usually caused by contaminated food, soil, water, and other sources. The infections by travel-related or foodborne transmission can be the reason for serotype discrepancy between stream and clinical isolates. However, this was not confirmed because we could not review the patient’s medical records. This is a limitation of this study.

*S.* I 4,[5]12:i:-, which is a monophasic variant of *S.* Typhimurium, has emerged as a major global cause of human salmonellosis, and is often associated with multidrug resistance in clinical settings [1,22]. Our results confirm that *S.* I 4,[5]12:i:- strains from stream is closely related to clinical strains (high similarity of >90%), and it exhibits moderate resistance to ciprofloxacin. This indicates that attention should be paid to preventing the transmission of this serotype through the stream environment.

Surprisingly, we observed 100% PFGE homology between the stream and clinical isolates of *S.* Mbandaka, *S.* Infantis, and *S.* Livingstone. Although *S.* Livingstone is relatively rare, it has been implicated in nosocomial outbreaks [23]. Our study confirmed, by WGS, that both the stream and clinical strains belonged to ST543. These results are consistent with previous reports showing that all five *S.* Livingstone-induced invasive infections belong to clade-5 [ST543] [24]. In particular, the authors reported that the proportions of clade-5 (ST543) increased from 23.4% (before 2010) to 54.3% (after 2010) in multidrug-resistant *S.* Livingstone. This observation underscores the potential public health significance of streamborne *S.* Livingstone. Similarly, *S.* Mbandaka showed the same MLST profiles for both stream and clinical strains, and all strains were identified as ST413. Showing the same genotype between stream and clinical strains indicates that stream environments are closely related to human infection. Although the current prevalence of *S.* Mbandaka may be low in Republic of Korea, outbreaks caused by ST413 have been reported in the European Union and United Kingdom [4]. Therefore, continuous surveillance is necessary to reduce the risk of ST413 outbreaks.

In this study, the resistance rates to antimicrobials were similar in stream and clinical strains. However, a notable difference is that the resistance rate of SXT was higher in stream strains (27.3%) than in clinical strains (6%). *S.* Typhimurium strains from streams showed high resistance rates to antimicrobials. Specifically, 20 (87.0%) of 23 *S.* Typhimurium were resistant to ampicillin, chloramphenicol, and SXT. This means that these are multi-drug resistant. Callahan et al. reported that antimicrobial resistance of Salmonella isolated from rivers in the United States [8]. They found that *S.* Typhimurium exhibited high antimicrobial resistance. This result is very similar to our data.

There is only one isolate exhibiting resistance to cefotaxime in our study. CTX-M is a major type of ESBL enzyme leading to resistance to the beta-lactam antimicrobial. It has been known that common CTX-Ms of *Salmonella* from clinical isolates were CTX-M-15, CTX-M-55 [25]. However, there is no *Salmonella* strain harboring the *bla*_CTX-M_ gene in this study. The other resistance mechanisms of cefotaxime were not determined in this study. In addition, we did not conduct additional investigations on clinical isolates because all isolates from the stream were negative for the *bla*_CTX-M_ gene. This is also a limitation of this study. One noteworthy finding is the high rate of intermediate resistance to ciprofloxacin observed in stream isolates. A high prevalence of intermediate resistance to ciprofloxacin in clinical isolates was shown in our previous study [1]. This could be a potential risk leading to the resistance to ciprofloxacin.

## 5. Conclusions

We detected 23 serotypes of *Salmonella* from stream environments in Republic of Korea. Among the strains, 73.9% of the serotypes (N = 17) were isolated from both the streams and patients. The most common serotypes were *S.* Typhimurium, *S.* Livingstone, *S.* Infantis, *S.* I 4,[5]12:i:-, and *S.* Thompson. *S.* Mbandaka and *S.* Livingstone isolates from streams were identified as ST413 and ST543, respectively, demonstrating clonal identity with isolates from clinical specimens. Our findings suggest that isolates from stream environments are closely related to patient isolates. Therefore, continuous monitoring of stream environments is essential to control the spread of *Salmonella* infection.

## Figures and Tables

**Table 1:** Serotype distribution of *Salmonella* isolates from streams.

Serogroup B (N = 37)			Serogroup C (N = 59)			Serogroup D (N = 2)		
Serotype	No.	%	Serotype	No.	%	Serotype	No.	%
*S.* Typhimurium	23	20.9%	*S.* Livingstone	19	17.27%	*S.* Panama	2	1.8%
*S.* I 4,[5],12:i:-	6	5.5%	*S.* Infantis	12	10.91%	**Serogroup E (N = 1)**		
*S.* Saintpaul	4	3.6%	*S.* Othmarschen	7	6.36%	**Serotype**	**No.**	**%**
*S.* Agona	3	2.7%	*S.* Thompson	6	5.45%	*S.* Senftenberg	1	0.9%
*S.* Stanley	1	0.9%	*S.* Montevideo	4	3.64%	**Serogroup G (N = 8)**		
			*S.* Bareilly	3	2.73%	**Serotype**	**No.**	**%**
			*S.* Narashino	2	1.82%	*S.* Worthington	8	7.3%
			*S.* Albany	1	0.91%	**Serogroup V (N = 2)**		
			*S.* Braenderup	1	0.91%	**Serotype**	**No.**	**%**
			*S.* Kentucky	1	0.91%	IV 44:z4,z24:/Christiansborg/IIIa 44:z4,z24:-	2	1.8%
			*S.* Mbandaka	1	0.91%	**Serogroup Y (N = 1)**		
			*S.* Rissen	1	0.91%	**Serotype**	**No.**	**%**
			*S.* Narashino/II 6,8:a:e,n,x	1	0.91%	IIIb48:k:z	1	0.9%

**Table 2:** Antimicrobial susceptibility results of *Salmonella* serotype isolates in streams and clinical samples.

Antimicrobial Agents	Stream (%)			Clinical (%)		
	S	I	R	S	I	R
Ampicillin	72.7	0	27.3	80.7	0	19.3
Azithromycin	100	0	0	100	0	0
Chloramphenicol	80.9	0	19.1	88	2	10
Cefotaxime	99.1	0	0.9	97.2	0.4	2.4
Ciprofloxacin	73.6	26.4	0	77.1	20.5	2.4
Trimethoprime-sulfamethoxazole	72.7	0	27.3	93.2	0.8	6
Tetracycline	79.1	0	20.9	82.3	0	17.7
Imipenem	100	0	0	100	0	0

**Table 3:** Quinolone resistance-determining region mutations of *Salmonella* from streams.

Serotype	*gyrA*	*parC*	No.
Othmarschen	D87G	T57S	1
I 4,[5],12:i:-	D87N	No mutation	6
Agona	No mutation	T57S	1
Senftenberg	No mutation	T57S	1
Albany	S83F	T57S	1
Total			10

**Table 4:** Genetic profiles of *S.* Mbandaka and *S.* Livingstone by WGS.

Serotype	Origin	Genome Size (bp)	Contigs	Percent G + C	No. Proteins	MLST	cgMLST	% Allele Matches
*S.* Mbandaka	Stream	4,659,350	9	52.22	4332	413	110,743	91.91
	Patient	4,809,463	7	52.06	4502	413	123,743	91.84
	Patient	4,648,559	12	52.22	4333	413	110,743	91.97
	Patient	4,648,996	13	52.22	4333	413	110,743	91.94
	Patient	4,648,490	12	52.22	4332	413	110,743	91.94
	Patient	4,655,192	10	52.22	4334	413	110,743	91.91
*S.* Livingstone	Stream	4,702,613	12	52.18	4375	543	73,367	91.64
	Patient	4,707,635	11	52.16	4369	543	73,367	91.54
	Patient	4,692,629	9	52.16	4359	543	73,367	91.57

## Data Availability

The original contributions presented in this study are included in the article. Further inquiries can be directed to the corresponding author.
